# A case report of a gastrobronchial fistula and lung abscess caused by leakage from the staple line of a gastric tube after esophagectomy for esophageal cancer

**DOI:** 10.1186/s40792-021-01178-8

**Published:** 2021-04-15

**Authors:** Tohru Nishimura, Chisakou Fuse, Masayuki Akita, Nobuhisa Takase, Eri Maeda, Koichiro Abe, Akihito Kozuki, Kunio Yokoyama, Tomohiro Tanaka, Shinji Kishi, Toshihiko Sakamoto, Tetsuya Sakai, Kunihiko Kaneda

**Affiliations:** 1 Department of Surgery, Kakogawa Central City Hospital, Kakogawa, 675-8611 Japan; 2 Department of Thoracic Surgery, Steel Memorial Hirohata Hospital, Himeji, Japan; 3 Department of Surgery, Steel Memorial Hirohata Hospital, Himeji, Japan

**Keywords:** Gastrobronchial fistula, Esophagectomy, Anastomotic leakage, Esophageal cancer, Intercostal muscle flap

## Abstract

**Background:**

Gastrobronchial fistulas are rare, but life-threatening, complications of esophagectomy. They are caused by anastomotic leakage and mainly occur around anastomotic sites. In the present paper, we report a rare case of leakage from the staple line of a gastric tube after esophagectomy for esophageal cancer, which was successfully treated using an intercostal muscle flap and lung resection.

**Case presentation:**

A 61-year-old male underwent subtotal esophagectomy with regional lymphadenectomy for esophageal cancer. The sutures along the staple line of the gastric tube failed 11 days after surgery, and a pulmonary abscess was also found on imaging. The abscess did not heal after conservative treatment; therefore, right lower lobectomy, gastrobronchial fistula resection, primary closure, and patching of the leaking portion of the gastric tube with an intercostal muscle flap were performed 9 months after the first operation. The patient’s postoperative course was uneventful, and he was discharged on the 354th day.

**Conclusions:**

We experienced a case involving a gastrobronchial fistula caused by leakage from the staple line of a gastric tube and successfully treated it by performing right lower lobectomy and patching the leak with an intercostal muscle flap.

## Background

Surgical treatment for esophageal cancer is highly invasive because it requires an extensive operation, involving the neck, chest, and abdomen. Various postoperative complications are often observed, including anastomotic leakage, pneumonia, the accumulation of pleural effusion, and gastric stasis as early postoperative complications and anastomotic stricture as a late complication, which require intensive care to resolve [1–3].

Gastrobronchial fistulas are rare, but life-threatening, complications of esophagectomy. They are caused by anastomotic leakage and mainly occur around anastomotic sites. The treatment strategy for gastrobronchial fistulas is determined by the location and size of the fistula and the patient's condition. Although conservative treatment and bronchoscopic or endoscopic closure can be effective for patients whose condition is stable, surgical interventions are often required for those in unstable conditions [4–6].

In the present paper, we report a gastrobronchial fistula and lung abscess caused by leakage from the staple line of a gastric tube. These complications were successfully treated using right lower lobectomy and an intercostal muscle flap.

## Case presentation

The patient was a 61-year-old male, who was brought to our hospital with severe upper abdominal pain. He had a history of gastric perforation caused by a benign ulcer and had undergone an operation for it 10 years earlier. The computed tomography (CT) images obtained at presentation revealed recurrent gastric perforation with free air around the stomach. Thus, omental patching was performed emergently for the gastric perforation, which affected the posterior wall of the lower gastric body.

The patient’s postoperative course was uneventful. However, esophagogastroduodenoscopy conducted one month after the surgery showed an ulcer scar in the stomach and an irregular mucosa in the lower thoracic esophagus, which was diagnosed as squamous cell carcinoma based on a biopsy examination.

We performed transthoracic subtotal esophagectomy with regional lymphadenectomy in the left lateral recumbent position. The reconstruction involved esophagogastric tube anastomosis in the thoracic cavity via the posterior mediastinal route. The gastric tube was created so that it avoided the ulcer site, and no blood flow insufficiency was observed. The esophageal cancer was diagnosed as T1b N0 M0, according to the UICC-TNM classification 7th edition.

**Fig. 1:**
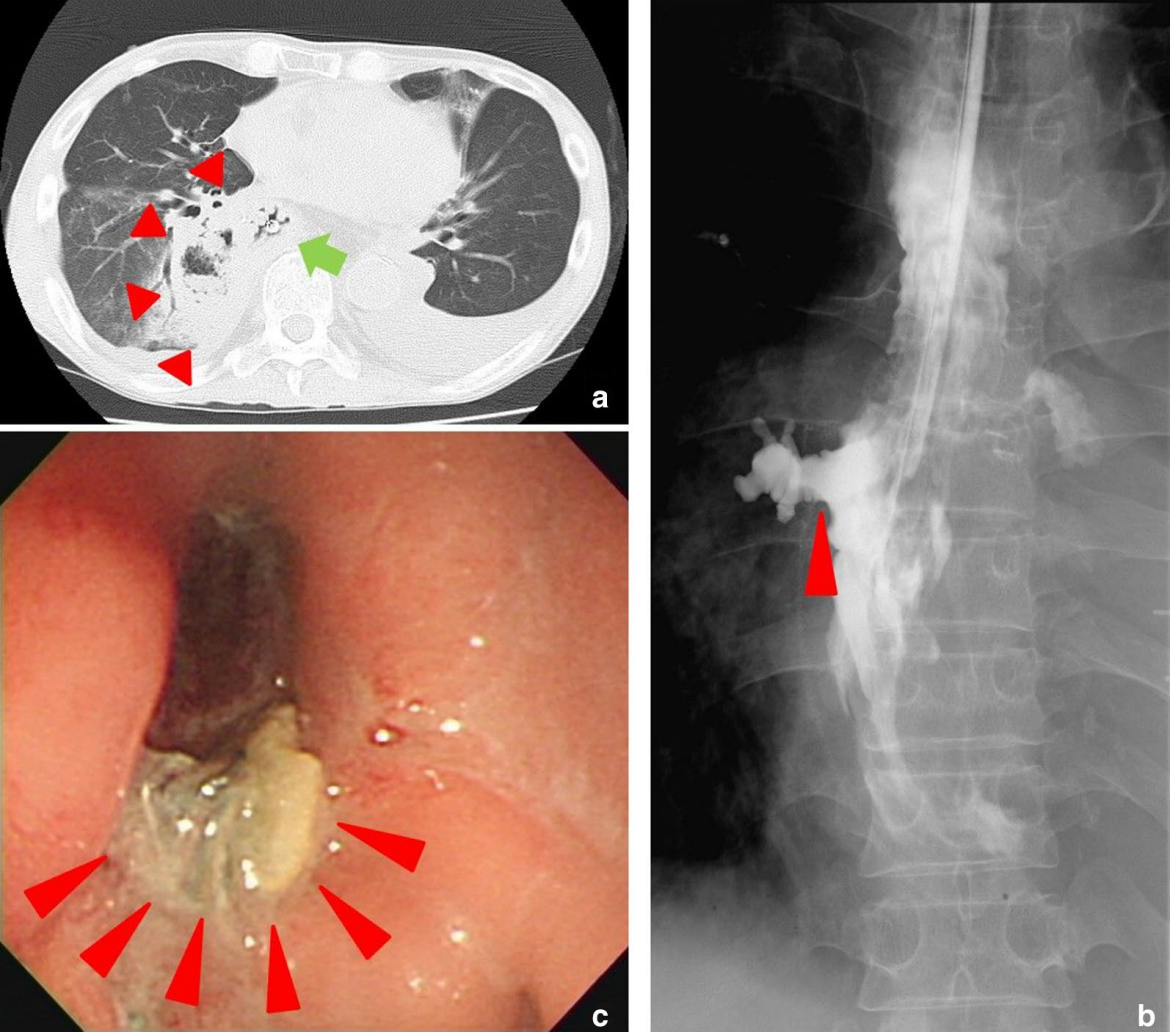
Imaging examinations of the fistula and pulmonary abscess performed before the conservative treatment. **a** A CT image showed a cavity with an air bubble on the right side of the gastric staple line. The green arrow indicates the gastric tube, and the red arrowheads show the abscess. **b** A Gastrografin esophagram showed extravasation into the right side of the thoracic cavity (red arrowheads). **c** An endoscopic examination confirmed that the fistula was located on the anal side of the anastomotic site. The red arrowheads indicate the fistula

**Fig. 2:**
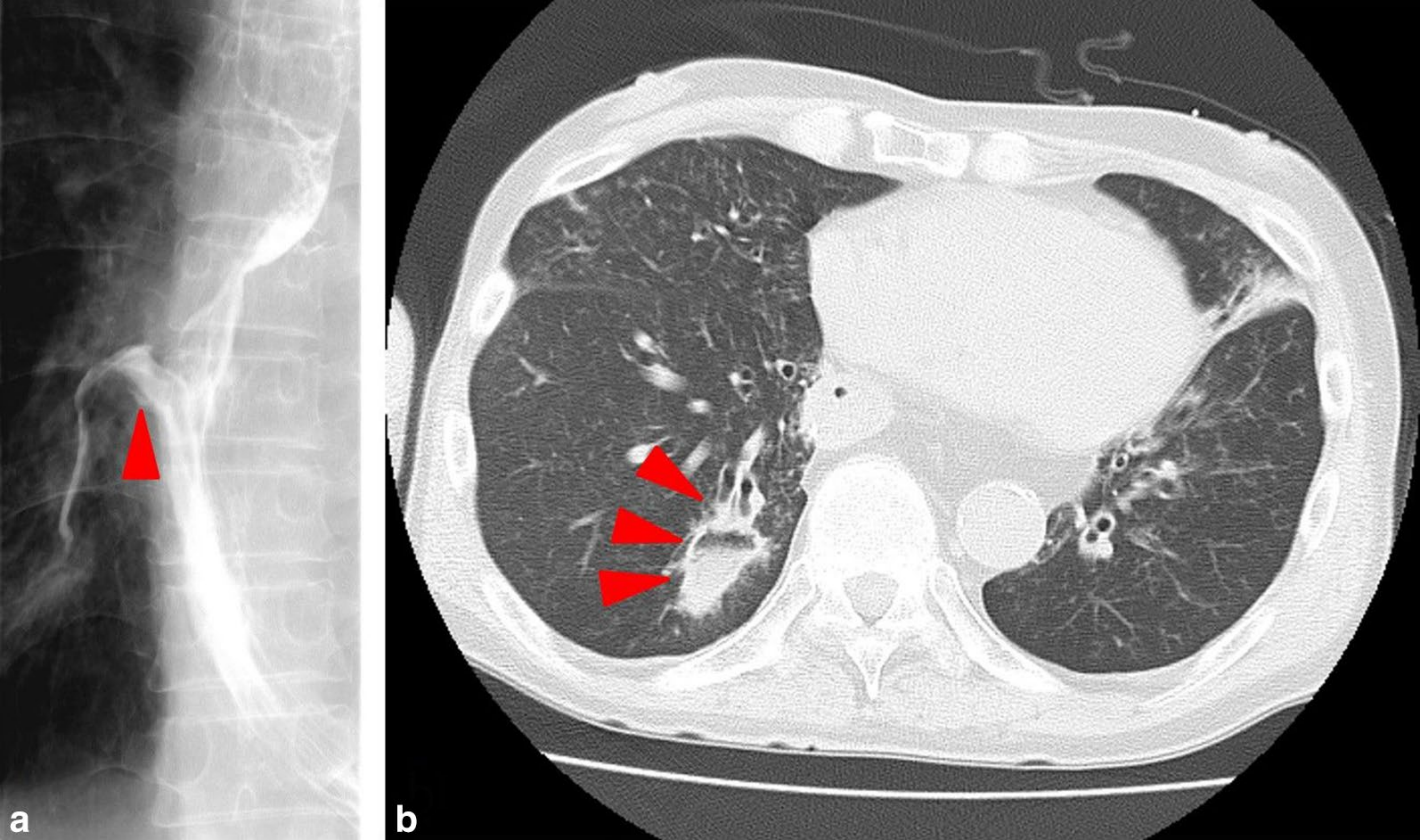
Imaging examinations performed after the conservative treatment. **a** A Gastrografin esophagram showed that the leakage was localized (red arrowhead). **b** CT showed an abscess as a reduction in permeability localized in the right lung (red arrowheads)

Eleven days after the surgery, the patient developed a fever and complained of chest and abdominal tightening and pain. CT showed a right-sided intrathoracic abscess (Fig. 1a). In addition, gastrointestinal fluoroscopy revealed leakage from the staple line of the gastric tube (Fig. 1b). An endoscopic examination confirmed the presence of a fistula on the anal side of the anastomotic site (Fig. 1c). The patient was treated with antibiotics, fasting, and a high-caloric infusion, resulting in a gradual recovery (Fig. 2). Sixty-eight days after the esophagectomy, the re-start of oral intake caused the right thoracic abscess to worsen. Thus, a nasotracheal tube was used to drain the chest abscess, and enteral feeding and intravenous hyperalimentation were initiated. However, the patient did not completely recover.

**Fig. 3:**
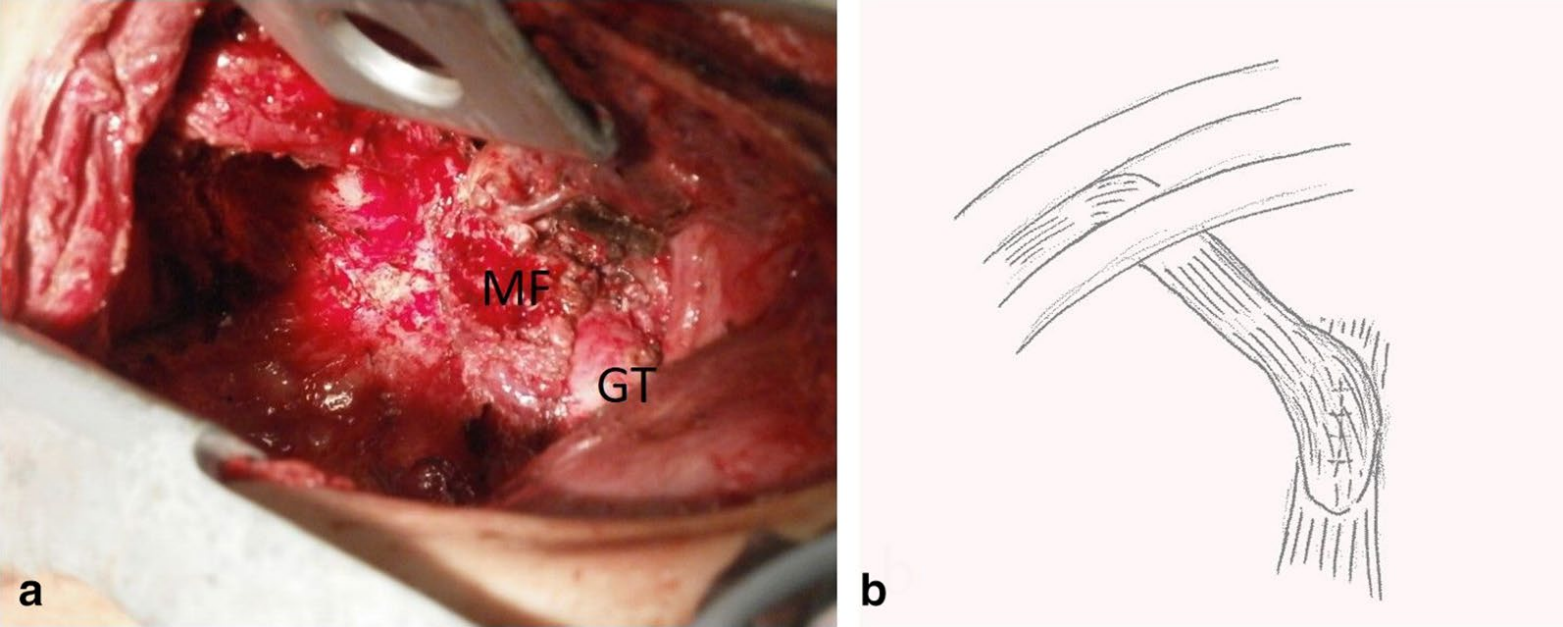
Intraoperative images. **a** The right lower pulmonary lobe was excised, and an intercostal muscle flap was placed over the suture line of the conduit. *GT* gastric tube, *MF* muscle flap. **b** The schema of the surgery is shown

Bronchoscopy identified a fistula, extending from the right B6 bronchus to the right lung abscess. Therefore, the patient underwent right lower lobectomy, gastrobronchial fistula resection, primary closure of the fistula, and patching of the leak in the gastric tube with an intercostal muscle flap 9 months after the initial surgery. Since the 5th intercostal muscle, which had been opened in the previous operation, had thinned, the chest was opened along the upper edge of the 7th rib, and the 6th intercostal muscle was dissected along the 6th rib to create a muscle flap. The locations of the lung abscess and fistula could not be confirmed, and lower lobe resection was conducted. The lower lobe branch of the pulmonary artery was ligated and dissected, and the pulmonary vein and bronchus were dissected with a stapler. The fistula was dissected along the gastric tube to complete the lower lobectomy. The fistula opening measured 2.5 cm in diameter, and no blood flow insufficiency was observed in the mucosa or serosa. The fistula was closed by suturing it directly in the longitudinal direction with 3-0 absorbent thread, a serosal muscular layer suture was added, and the fistula was covered and fixed in place with an intercostal muscle flap (Fig. 3). After the operation, the patient recovered completely. No recurrence has been seen in the year since the initial surgery.

## Discussion

In esophagectomy for patients with esophageal cancer, the stomach is the most commonly used esophageal substitute. Among the various complications associated with gastric tubes after subtotal esophagectomy, anastomotic leakage is the most frequent. Fistulas between the airway and gastric tube are rare, but potentially life-threatening complications of such surgery [1–3]. The frequency of gastrobronchial fistulas after esophagectomy was reported to range from 0.04 to 0.3% [3, 7]. It produces various symptoms, including coughing during ingestion in mild cases, and recurrent bronchopneumonia or mediastinitis in severe cases. X-ray fluoroscopic examinations are useful for detecting gastrobronchial fistulas and determining the locations of any leaks. Endoscopy and bronchoscopy can also be helpful for identifying the locations of fistulas. Although endoscopic closure is the only possible cure, surgical interventions including the closing of tracheal and esophageal defects and the filling of abscess cavities with fatty tissue or a muscle flap is often necessary [3, 6] (Table 1).

**Table 1:** Previously reported treatment methods of gastrobronchial fistula

*Endoscopic obliteration*
Fast-hardening amino acid solution [8]
Fibrin glue [6, 9]
Self-expanding stent [5, 10]
Dual self-expandable stent [11]
Placement of a cardial septal occluder [12]
Titanium clips [13]
*Surgical intervention*
Direct approach
Reconstruction by colonic interposition [6]
Reconstruction by jejunum [14]
Pedicled pericardial flap [15, 16]
Omental patch, pleural patch [6]
Muscle flap
Pectoralis major muscle flap (PMM) [17]
Latissimus dorsi myocutaneous flap [15]
Intercostal muscle [4, 6, 18]
Alloderm patch with intercostal muscle flap [19]
Lobectomy [20]
Lobectomy and intercostal muscle patch

Bronchoesophageal fistulas that arise after esophagectomy usually occur around the anastomosis between the esophagus and the stomach following anastomotic leakage, but in rare cases they can occur after leakage from the staple line of a gastric tube [17]. Gastrobronchial fistulas that arose along the staple lines of gastric tubes have been reported as late complications caused by gastric ulcers, chronic erosion along the staple line, or compression due to long-term feeding tube insertion [7]. However, there have been some reports about fistulas that occurred in the early phase after esophagectomy [10, 18] (Table 2). Reames et al. reported a case in which a fistula occurred along a gastric conduit staple line, which was successfully repaired using an acellular dermal matrix patch (AlloDerm, LifeCell, Inc., Branchburg, NJ) reinforced with an intercostal muscle flap [14]. Buskens et al. studied the cases of 383 patients that underwent subtotal esophagectomy and reported that 6 patients developed gastrobronchial fistulas. Among these cases, one fistula was caused by leakage along the staple line of a gastric tube. This patient had mild mediastinitis and respiratory insufficiency. A cervical esophagostomy was created, while the vital gastric tube was left in situ. A drainage tube was inserted through the neck into the gastric tube to flush the mediastinal cavity, and the patient recovered quickly [6]. Pramesh et al. reported the case of a patient who underwent transthoracic esophagectomy and developed a gastrobronchial fistula caused by exposed staples eroding the right bronchus [18]. The patient was successfully treated with re-exploratory thoracotomy and direct closure of the fistula. Meyerson et al. reported that many patients with gastrobronchial fistulas needed to undergo surgical repair, fistula closure, and vascularized tissue grafting to minimize recurrence [3].

**Table 2:** Previously reported cases with gastrobronchial fistula caused by leakage from the staple line of a gastric tube after resection for esophageal cancer

Case	Age	Sex	Tumor location	Stage	Reconstruction	Location of fistula	Postoperative days	Symptom	Treatment
1 [6]	61	F	–	–	Posterior mediastinum	Trachea	–	Mild mediastinitis, Respiratory insufficiency	Cervical esophagostomy and drainage, reconstruction by subcutaneous colonic interposition
2 [21]	55	M	Middle third	–	Posterior mediastinum	Right bronchus	10	Respiratory insufficiency	Re-exploratory thoracotomy
3 [19]	71	F	Middle	T1bN0	Posterior mediastinum	Right bronchus	–	None	Alloderm patch with intercostal muscle flap
Present case	61	M	Low	T1bN0	Posterior mediastinum	Right bronchus	11	Fever, chest and abdominal pains	Lobectomy and intercostal muscle flap

In the present case, the patient underwent transthoracic esophagectomy and developed a gastrobronchial fistula caused by leakage from the staple line of the gastric tube. We usually perform subtotal esophagectomy for lower esophageal cancer and esophagogastric anastomoses in the neck via the poststernal route. However, we considered that a gastric tube of sufficient length could not be obtained via this route in the current case due to the previous gastric ulcer; therefore, reconstruction was carried out via the posterior mediastinal route. The ulcer scar was avoided when the gastric tube was made, and it was visually confirmed that there was sufficient blood flow through the gastric tube. If we had been concerned about impaired blood flow through the gastric tube, we would have performed the reconstruction using the colon or small intestine. At the time the patient was treated, it was not possible to conduct blood flow assessments using indocyanine green or thermal imaging at our institution [22]. The initial non-surgical treatment almost closed the fistula, but the subsequent restarting of oral intake caused the fistula to worsen. Then, a lung abscess formed, and its drainage was considered to be a priority; therefore, endoscopic closure was not performed. If the lung abscess had not formed, then endoscopic treatment might have been sufficient. We continued administering a high-calorie infusion and enteral nutrition to improve the patient’s poor nutritional condition. However, the fistula did not close, and pulmonary resection to remove the lung abscess, direct closure of the fistula, and patching of the staple line with an intercostal muscle flap were performed. We considered that an intercostal muscle flap of sufficient volume could be harvested from the same surgical field, and hence, did not use a latissimus dorsi muscle or pectoralis major flap, which are often used in head and neck surgery.

There are many causes of gastric tube perforation, including gastric ulcers, chronic erosion of a staple line, compression caused by the long-term insertion of a feeding tube, impaired blood flow, an increase in intragastric pressure, or staple malformation. Baker et al. reported that leakage from the staple line due to ischemia usually occurs within 7 days after surgery [23]. In the present case, the gastric tube perforation occurred 11 days after surgery, and impaired blood flow through the gastric tube was not observed, even at the time of the reoperation. He consumed all the food provided, it might have been caused by an increase in intragastric pressure. We usually reinforce the staple line of the gastric tube with serosal muscle layer sutures, and perform pyloric digital dilatation.

Gastrobronchial fistulas caused by leakage from a staple line differ from fistulas caused by anastomotic leakage. The staple lines of gastric tubes run on the lesser side of the conduit, and leakage occurs on the anal side of anastomotic leaks. Therefore, leakage from the staple line causes lower mediastinitis, which can lead to fistulas developing in the lower lung or bronchus. The direct closure of a fistula via bronchoscopy is difficult. Hong Kwan et al. reported that benign bronchoesophageal fistulas could be successfully treated via lung lobectomy [16]. To the best of our knowledge, this is the first reported case in which lobectomy was used to manage a gastrobronchial fistula caused by leakage from the staple line of a gastric tube.

## Conclusion

We experienced a case of a gastrobronchial fistula caused by leakage from the staple line of a gastric tube and successfully treated it by performing pneumectomy and patching the leak using an intercostal muscle flap. Gastrobronchial fistulas are rare, but in cases in which minimally invasive treatment does not help to close the fistula, a reoperation involving excision of the infected lung can bring about prompt healing.

## Data Availability

Not applicable.
